# Supplementary material for: New approach for FIB-preparation of atom probe specimens for aluminum alloys
Source: PLoS One. 2020 Apr 2;15(4):e0231179. doi: 10.1371/journal.pone.0231179 (PMC7117760; doi:10.1371/journal.pone.0231179)
Supplement: S1 Fig — (a) Atomic reconstruction and (b) composition profile along the arrow in (a) for the Gallium showing a composition gradient from the top of the specimen to the bottom. (c) Corresponding mass spectrum of the dataset, that was cut at 100 Da (no peaks are observed at larger Da). The inset shows a zoom of the region between 64 and 74 Da, with the 69Ga+ peak and a much smaller 71Ga+ peak. The little peak at 65 Da corresponds to Cu, which is a classical impurity found in low concentrations. (PDF) [file pone.0231179.s001.pdf]

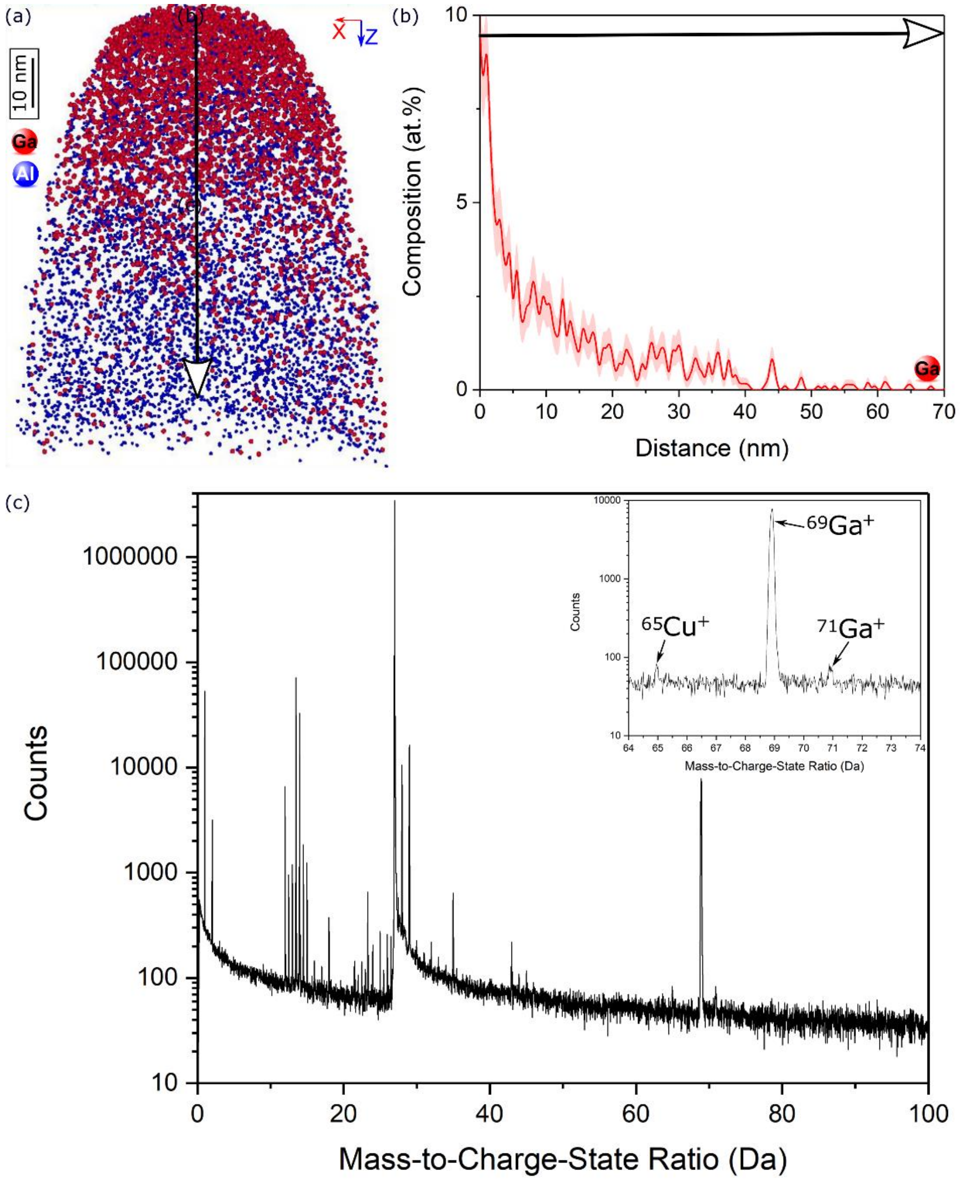

S1 Fig: Ga implantation during milling for the cryo-prepared specimen.
(a) Atomic reconstruction and (b) composition profile along the arrow in (a) for the Gallium showing a composition gradient from the top of the specimen to the bottom. (c) Corresponding mass spectrum of the dataset, that was cut at 100 Da (no peaks are observed at larger Da). The inset shows a zoom of the region between 64 and 74 Da, with the 69Ga+ peak and a much smaller 71Ga+ peak. The little peak at 65 Da corresponds to Cu, which is a classical impurity found in low concentrations
