# Supplementary material for: New approach for FIB-preparation of atom probe specimens for aluminum alloys
Source: PLoS One. 2020 Apr 2;15(4):e0231179. doi: 10.1371/journal.pone.0231179 (PMC7117760; doi:10.1371/journal.pone.0231179)
Supplement: Attachment — Submitted filename: response to reviewers-1.docx [file pone.0231179.s003.docx]

IRCP

11 rue Pierre et Marie Curie

75005 Paris

Email of the corresponding author: l.lilensten@mpie.de

March 13^th^ , 2020

Dear Editor,

We are thankful to the reviewers for the minor revisions on our manuscript entitled **“New approach for FIB-preparation of atom probe specimens for aluminum alloys”** (manuscript number PONE-D-19-33571). In the following, we listed the reviewers’ comments & questions as well as our answers. The Reviewers’ comments are italicized, and our modifications to the manuscript’s main text are in bold. The quotations from the main text are written in blue.

**Reviewer #1:**

*The authors have addressed the raised points, besides some minor points, to satisfaction of the reviewer.

Minor points:
- Something went wrong in labeling the poles, please check and correct Figure 1 b) and Figure 2 b).*

The two figures have been double checked and, indeed, corrected as follows:

Figure 1

Figure 2

*- There is a display error in the bibliography, reference [25]. "$\ensuremath\Sigma5$"*

It has been corrected.

**Reviewer #2:**

*The authors have addressed my points and the paper can be published after minor correction.

I am not fully convinced by Fig. 1. For me the difference between 1b and 1c is not totally clear. This could also be the same orientation. Maybe the authors can add a comment.*

The text was modified as follows:

“As highlighted by the red dashed circles, **it appears that there is a shift of the position of the main poles in Fig 1b. This is an indication that the upper and lower parts, on either side of the Ga-rich region, have different crystallographic orientation**, and this information suggest that the dense Ga region corresponds to a grain boundary.”
